# Efficient manipulation of gene expression using *Natronobacterium gregoryi* Argonaute in zebrafish

**DOI:** 10.1186/s12915-023-01599-x

**Published:** 2023-04-24

**Authors:** Zhangji Dong, Xu Chen, Run Zhuo, Yuanyuan Li, Zhihao Zhou, Ying Sun, Yan Liu, Mei Liu

**Affiliations:** Key Laboratory of Neuroregeneration of Jiangsu and Ministry of Education, NMPA Key Laboratory for Research and Evaluation of Tissue Engineering Technology Products, Co-Innovation Center of Neuroregeneration, Nantong University, Nantong Jiangsu, 226001 China

**Keywords:** NgAgo, Morpholino oligonucleotides, gDNA, Knockdown, Phenotype

## Abstract

**Background:**

*Natronobacterium gregoryi* Argonaute (NgAgo) was found to reduce mRNA without generating detectable DNA double-strand breaks in a couple of endogenous genes in zebrafish, suggesting its potential as a tool for gene knockdown. However, little is known about how it interacts with nucleic acid molecules to interfere with gene expression.

**Results:**

In this study, we first confirmed that coinjection of NgAgo and gDNA downregulated target genes, generated gene-specific phenotypes and verified some factors (including 5’ phosphorylation, GC ratio, and target positions) of gDNAs affecting gene downregulation. Therein, the sense and antisense gDNAs were equally effective, suggesting that NgAgo possibly binds to DNA. NgAgo-VP64 with gDNAs targeting promoters upregulated the target genes, further providing evidence that NgAgo interacts with genomic DNA and controls gene transcription. Finally, we explain the downregulation of NgAgo/gDNA target genes by interference with the process of gene transcription, which differs from that of morpholino oligonucleotides.

**Conclusions:**

The present study provides conclusions that NgAgo may target genomic DNA and that target positions and the gDNA GC ratio influence its regulation efficiency.

**Supplementary Information:**

The online version contains supplementary material available at 10.1186/s12915-023-01599-x.

## Background

Gene manipulation has been successfully practiced using engineered endonuclease techniques, including zinc-finger nucleases (ZFN) [1], transcription activator-like effector nucleases (TALEN) [2], and clustered regularly interspaced short palindromic repeats/CRISPR-associated protein 9 (CRISPR/Cas9) [3]. The Argonaute proteins are a family of endonucleases that use 5’-phosphorylated short single-stranded DNA as a guide for binding and cutting targets [4–7]. Similar to Cas9, Argonautes play a key role in gene expression suppression and defense against foreign nucleic acids [8, 9]. In subsequent studies, Gao et al. reported using *Natronobacterium gregoryi* Argonaute (NgAgo) to cleave plasmids and endogenous mammalian genes [10]. The DNase activity in NgAgo was not reproduced by others thereafter [11], but NgAgo was demonstrated to downregulate *fabp11a* and *ta* expression with a 5’ phosphorylated guide DNA (5’-p-gDNA) after delivery into zebrafish embryos by microinjection; also, it was noticed that a length of gDNA between 20 and 24 was preferred, and the downregulation effect was independent of its catalytic activity [12]. Ye et al. demonstrated through in vitro experiments that gDNA does not require 5’ phosphorylation to be effective, and NgAgo still exhibits RNA endonuclease activity [13]; and a recent study identified the cleavage site [14]. However, whether NgAgo interacts with genomic DNA in eukaryotic cells remains elusive. In this study, we aimed to address the interaction between NgAgo and DNA.

## Results

### NgAgo/gDNA downregulates gene expression and phenocopies morphants

We first examined the efficiency of NgAgo/gDNA-mediated gene downregulation. We randomly selected genes in the zebrafish genome and designed gDNAs targeting the coding regions of these genes (Additional File 1: Table S1). After coinjection of gDNA and NgAgo mRNA, embryos were randomly collected for qRT‒PCR at 24 hpf. For most of these genes, NgAgo/gDNA could downregulate the mRNA level (Additional File 2: Fig. S1A), suggesting its effectiveness for a wide range of genes.

**Fig. 1:**
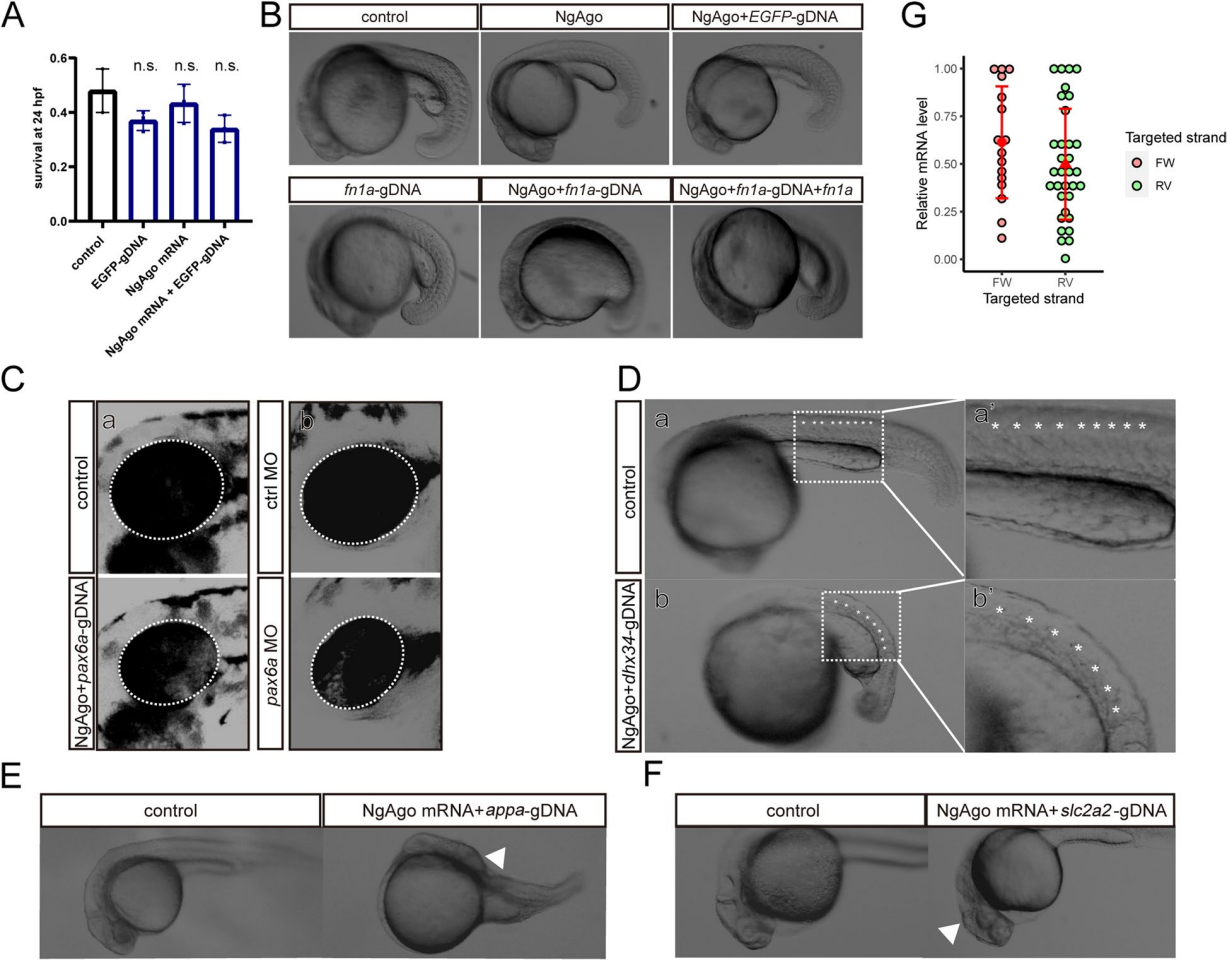
Downregulating gene expression using NgAgo/gDNA. **A** Survival rate of embryos after injection of gDNA and NgAgo mRNA. Experiments were performed in triplicate and repeated three times. The bars display mean ± se. Student’s* t* test was performed and n.s. indicates the difference is not statistically significant compared to control. *n* = 3 batches of injected embryos. **B** Specific alteration of morphology of zebrafish embryos after coinjection of NgAgo mRNA and *fn1a*-gDNA, observed at 22 hpf. The phenotype could be rescued by overexpressing *fn1a* mRNA. **C** Comparison of phonotypes generated by morpholino oligonucleotides and NgAgo/gDNA. (a) Representative NgAgo/*pax6a*-gDNA phenotype and (b) representative morphant phenotype of *pax6a* at 48 hpf compared to that of controls. **D** Phenotype of NgAgo-gDNA knockdown of *dhx34* in zebrafish at 24 hpf. (a) Wild-type phenotype. (b) Phenotype after NgAgo treatment. (a' and b’) are the enlarged images of the trunk in Panels a and b, and the asterisks (*) indicate the somites. **E**, **F** Phenotype of NgAgo-gDNA knockdown of *appa* (E) and *slc2a2* (F) in zebrafish at 36 hpf. The arrow indicates the malformed brain. **G** Statistical analysis of the knockdown efficiency of different targeted strands. *n* = 4

We observed whether NgAgo/gDNA induced potential toxicity and whether the phenotypes could be rescued by target gene reintroduction. First, we targeted *fn1a* with NgAgo/gDNA and compared the phenotype with control embryos that received only NgAgo mRNA, NgAgo with EGFP-gDNA, or only *fn1a*-gDNA, and a rescue group with additional *fn1a* mRNA. Injection of a nonspecific gDNA, NgAgo mRNA, or both did not significantly affect embryonic development or survival (Fig. 1A; Additional file 3: Table S2). Coinjection of NgAgo mRNA and *fn1a*-specific gDNA resulted in severe developmental abnormalities similar to a previous study [12], which could be rescued by 100 pg *fn1a* mRNA (Fig. 1B). At 48 hpf, zebrafish embryos that received NgAgo/gDNA targeting *pax6a* developed smaller eyes (Fig. 1Ca). The *pax6a* morphants (0.3 mM *pax6a* MO) similarly showed a small-eye phenotype (Fig. 1Cb). We also tested NgAgo/gDNA targeting *dhx34*, *appa*, and *slc2a2* (Fig. 1D-F). These phenotypes are consistent with reported results in morphants [15–17]. These results revealed that the potential toxicity of NgAgo/gDNA could be ignored and that its efficacy in downregulating gene expression is similar to that of MO.

Then, we further clarified that both phosphorylated and unphosphorylated gDNAs could efficiently downregulate gene expression (Additional File 2: Fig. S1AB; Additional File 1: Table S1, Additional File 4: Table S3), suggesting that 5’ phosphorylation in gDNAs is not a critical factor for the effectiveness of NgAgo, which is different from the case of PfAgo and TtAgo [6, 7]. Therefore, we used unphosphorylated gDNAs in this study. We also analyzed gDNAs targeting different strands. NgAgo mRNA with a sense or antisense gDNA was tested in zebrafish embryos. Interestingly, the results show that, on average, gDNAs targeting the sense and antisense strands are equally effective (Fig. 1G, Additional File 1: Table S1), suggesting possible binding of NgAgo/gDNA to genomic DNA.

### NgAgo/gDNA is tuned by the target position and GC content of gDNAs to effectively knock down target genes.

If NgAgo/gDNA targets genomic DNA to downregulate gene expression, it should, in some ways, work similarly to CRISPRi [18, 19]. As CRISPRi works more efficiently when targeted to the 5’ part of the transcribed region of a gene, we analyzed our dataset for the influence of target position on NgAgo knockdown efficiency. The difference in the position of the targeted loci may affect gene downregulation, and gDNAs targeted to the 5’ part of genes showed a more effective tendency (Additional File 2: Fig. S1C; Additional File 1: Table S1).

**Fig. 2:**
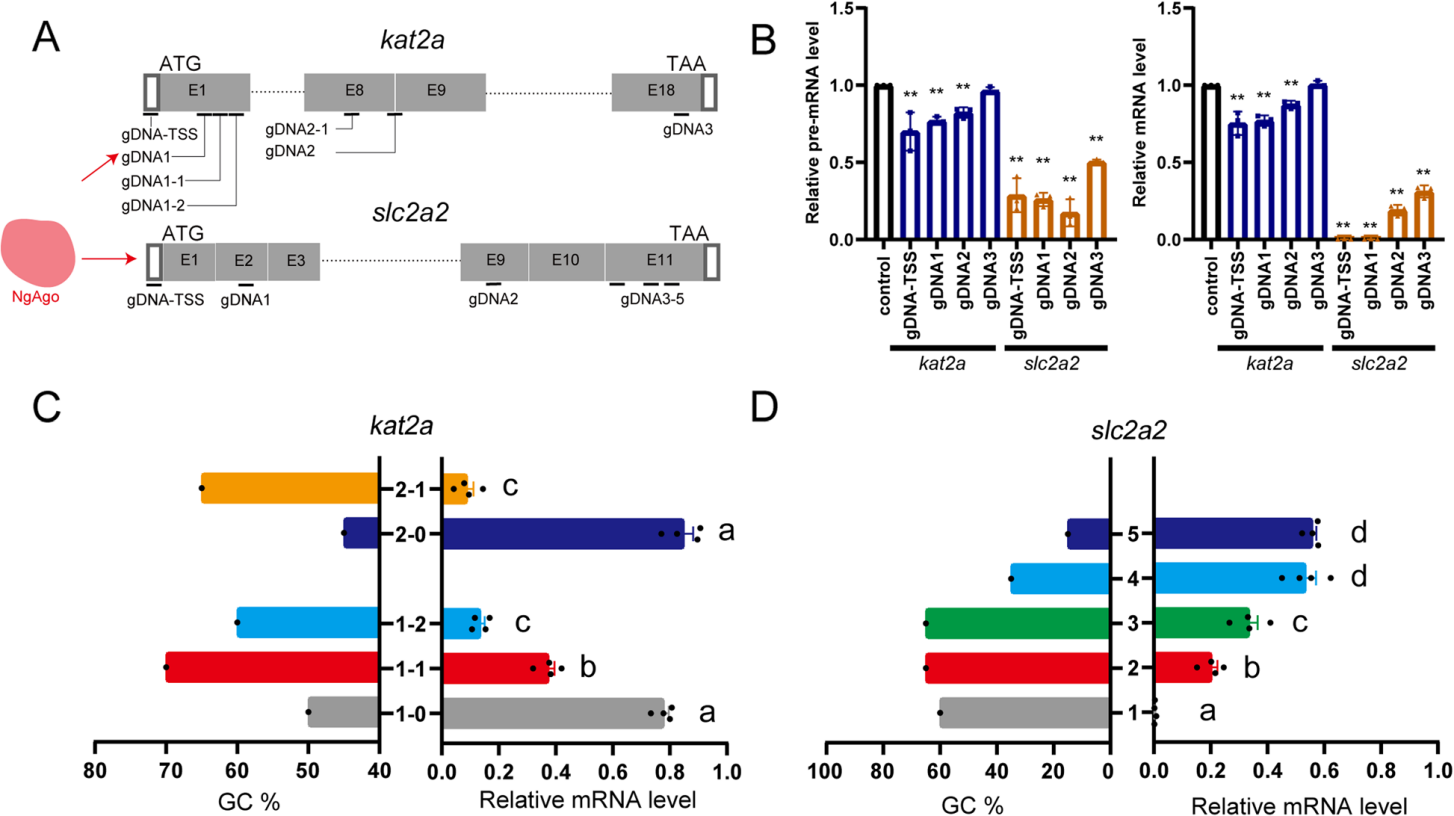
Tuning NgAgo/gDNA by target position and GC content of gDNAs to effectively knock down target genes. **A** Schematic diagram of gDNA design on *kat2a* and *slc2a2* in zebrafish. **B** Efficiency of downregulation of gDNAs at the 5’, middle and 3’ part of *kat2a* or *slc2a2 pre-mRNA and mRNA*, measured by qRT‒PCR at 24 hpf. qRT-PCR experiments were performed biologically three times. The bars display mean ± se. Student’s *t* test was performed and the ** indicated the difference compared to control is statistically significant (*p* < 0.01). *n* = 3. **C**, **D** The effect of the GC ratio of gDNAs targeting neighboring loci in *kat2a* and *slc2a2*. qRT-PCR experiments were performed biologically four times. The bars display mean ± se, and Student’s *t* test was performed and the different letters (a-d) indicate statistically significant differences (*P* < 0.05). *n* = 4

We then selected gDNAs for *kat2a*: *kat2a*-gDNA1, *kat2a*-gDNA2, and *kat2a*-gDNA3 (Additional File 1: Table S1), which were coinjected with NgAgo mRNA, and examined the efficiency of knockdown at 24 hpf. These gDNAs were targeted to loci at 1.8%, 48.28%, and 92.67% of the length of the mRNA, respectively, calculated with the transcription start site (TSS, targeted by gDNA-TSS) being 0% (Fig. 2A). The gDNA-TSS was designed to target the transcription start site (Fig. 2A). *kat2a*-gDNA2 was efficient in downregulating the level of *kat2a* mRNA, while *kat2a*-gDNA1 and *kat2a*-gDNA3 were less effective, and *kat2a-gDNA1* was weakly more effective than *kat2a*-gDNA3 (Fig. 2B, Additional File 5 Table S4). We selected *slc2a2* as another model gene (Additional File 1: Table S1). gDNAs were designed to target *slc2a2* at 2.7%, 38.2% and 56.4% of the length of the mRNA, respectively, and a gDNA-TSS targeting the TSS. We found that gDNAs targeting the 5’ part of genes were more potent in downregulating mRNA levels than those targeting the 3’ part (Fig. 2B, Additional File 6: Table S5). These results suggest that the position of a gDNA target is a determinant of the knockdown efficiency.

Additionally, we noticed in our dataset that the GC content of gDNA may affect its knockdown efficiency (Additional File 2: Fig. S1D), so we tested gDNAs of various GC contents. We injected NgAgo with the gDNAs *kat2a*-gDNA1, 1–1, and 1–2 targeting the 5’ part of *kat2a*, and *kat2a*-gDNA2 and 2–1 targeting the middle of *kat2a* (see sequences in Supp. Table 1), among which *kat2a*-gDNA1 (presented as 1–0 in Fig. 2C) and *kat2a*-gDNA2 (presented as 2–0 in Fig. 2D) had the lowest GC content in each cluster. The results showed that the activities of *kat2a*-gDNA1-1 and 1–2 were higher than that of gDNA1, while the activity of *kat2a*-gDNA2-1 was higher than that of gDNA2 (Fig. 2C, Additional File 7: Table S6). We also designed five gDNAs for *slc2a2* (*slc2a2*-gDNA1 to 5, Additional File 1: Table S1). The results showed that the gDNAs with higher GC content (1, 2, and 3) were significantly more efficient than those with lower GC content (4 and 5) (Fig. 2D, Additional File 8: Table S7).

### NgAgo/gDNA is mechanistically different from morpholino oligonucleotides knockdown

**Fig. 3:**
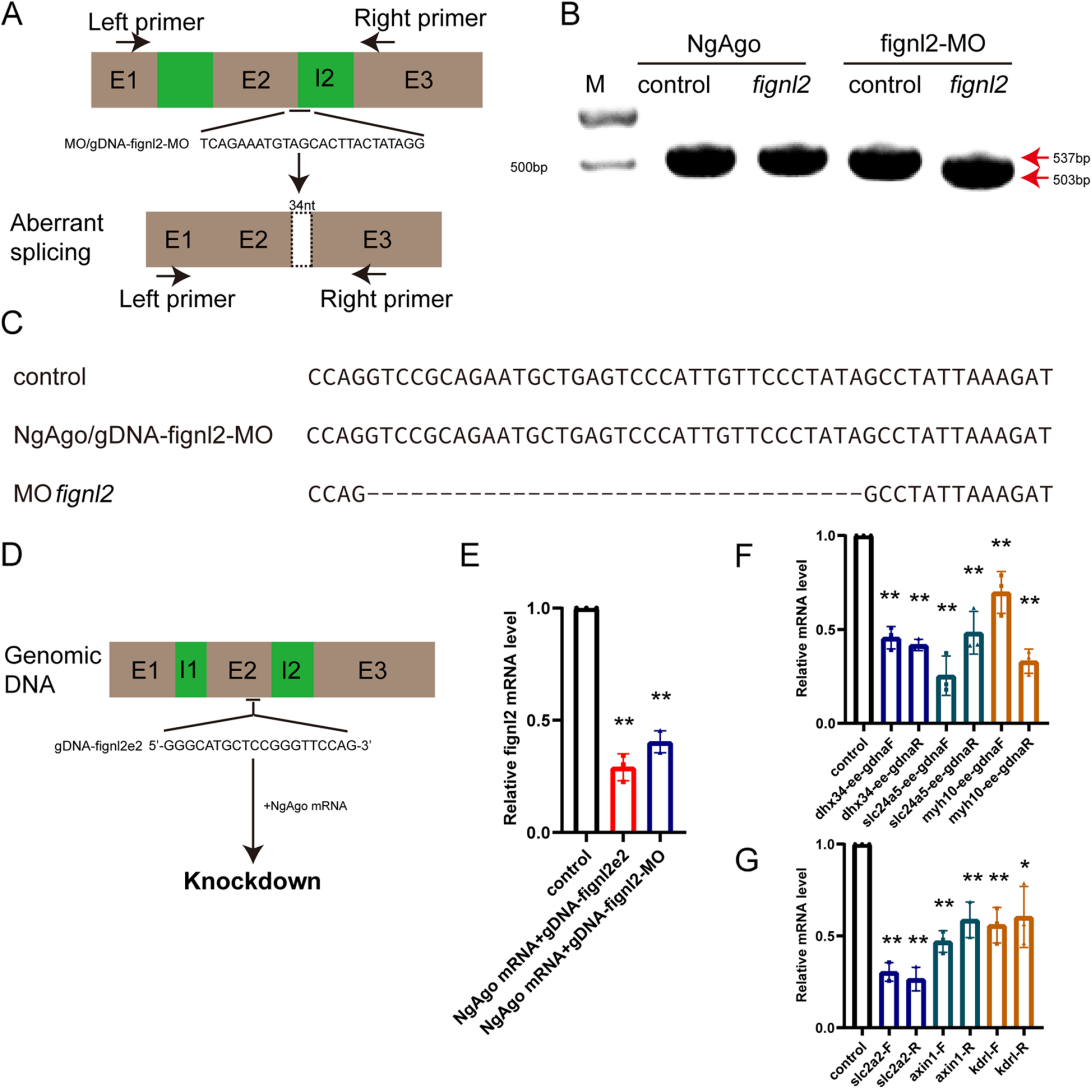
Comparison of the working models of MO and NgAgo. **A** Schematic diagram of morpholino oligonucleotide (MO)-induced *fignl2* knockdown causing aberrant splicing. **B** MO but not NgAgo treatment resulted in a change in the size of the *fignl2* RT‒PCR amplicon. The red arrow indicates the size of the PCR-amplified fragment. Control amplicon: 537 bp, amplicon from aberrant mRNA resulting from MO function: 503 bp. **C** MO treatment resulted in a 34-bp deletion in *fignl2* mRNA. No splicing changes were observed after NgAgo treatment. **D** Schematic diagram of NgAgo/gDNA targeting *fignl2* in exon 2. **E** *fignl2* mRNA level of 24 hpf embryos treated with NgAgo and gDNAs in (A) and (D). *n* = 3. **F** NgAgo-gDNA targeted to exon-exon boundaries reduced mRNA levels. *n* = 3. **G** NgAgo-gDNA targeted to intronic regions reduced mRNA levels. *n* = 3. The qRT‒PCR experiments were performed biologically three times. The bars display mean ± se. Student’s t test was performed and the ** and * indicate the difference compared to control is statistically significant (*p* < 0.01 and *p* < 0.05, respectively)

Furthermore, we tested whether NgAgo/gDNA shares the working mechanism of MOs. If so, we would expect changes in the size of the RT‒PCR amplicons of the target mRNAs. We designed an MO (fignl2-MO) targeting *fignl2* by blocking splicing and synthesized a gDNA of the identical sequence of fignl2-MO for NgAgo, named gDNA-fignl2-MO (Fig. 3A). After injection of fignl2-MO into 1-cell stage zebrafish embryos, we observed that MO disrupted the expression of *fignl2* (Fig. 3B, Additional File 9: Fig. S2). The fignl2-MO caused a 34 nt deletion in the mRNA (Fig. 3C). We coinjected NgAgo mRNA and gDNA-fignl2-MO to determine whether NgAgo-gDNA works like MO in terms of blocking splicing. The results showed that at the same target at the exon‒intron border, NgAgo/gDNA-fignl2-MO downregulated the expression of *fignl2*, but it did not alter the size of the amplicon containing the targeted region, and the sequence of the targeted region was not changed, as confirmed by Sanger sequencing (Fig. 3CE, Additional File 10: Table S8).

These results suggest that NgAgo/gDNA may not work in a splicing-blocking manner when targeted to exon‒intron boundaries. However, a reduction in mRNA level was seen in the NgAgo/gDNA-fignl2-MO-injected embryos, although the knockdown was not as effective as an exon-targeting gDNA, gDNA-fignl2e2, which targets Exon 2 (Fig. 3DE, Additional File 10: Table S8).

To answer whether NgAgo targets mRNA, we designed gDNAs targeting only mature mRNA at their exon‒exon boundaries (Fig. 3F, Additional File 11: Table S9, Additional File 12: Table S10) and found that these gDNAs effectively downregulated mRNA levels. We also tested knockdown using gDNAs targeted to intronic targets, which would be effective if gDNAs target molecules other than mRNA, e.g., pre-mRNA or genomic DNA (Supp. Table 1). The gDNAs targeting the introns of *slc2a2*, *axin1,* and *kdr1* were effective in downregulating mRNA levels regardless of whether they harbored sense or antisense strand sequences (Fig. 3G, Additional File 11: Table S9, Additional File 12: Table S10).

### NgAgo-VP64 targets genomic DNA and upregulates target gene expression

Our results above suggest the possibility of NgAgo/gDNA targeting genomic DNA in zebrafish. Recently, NgAgo was found to bind to and nick DNA in E. coli [20]. While our results above show similarities between NgAgo/gDNA and CRISPRi, it remains to be answered whether NgAgo-gDNA is capable of targeting eukaryotic genomic DNA.

**Fig. 4:**
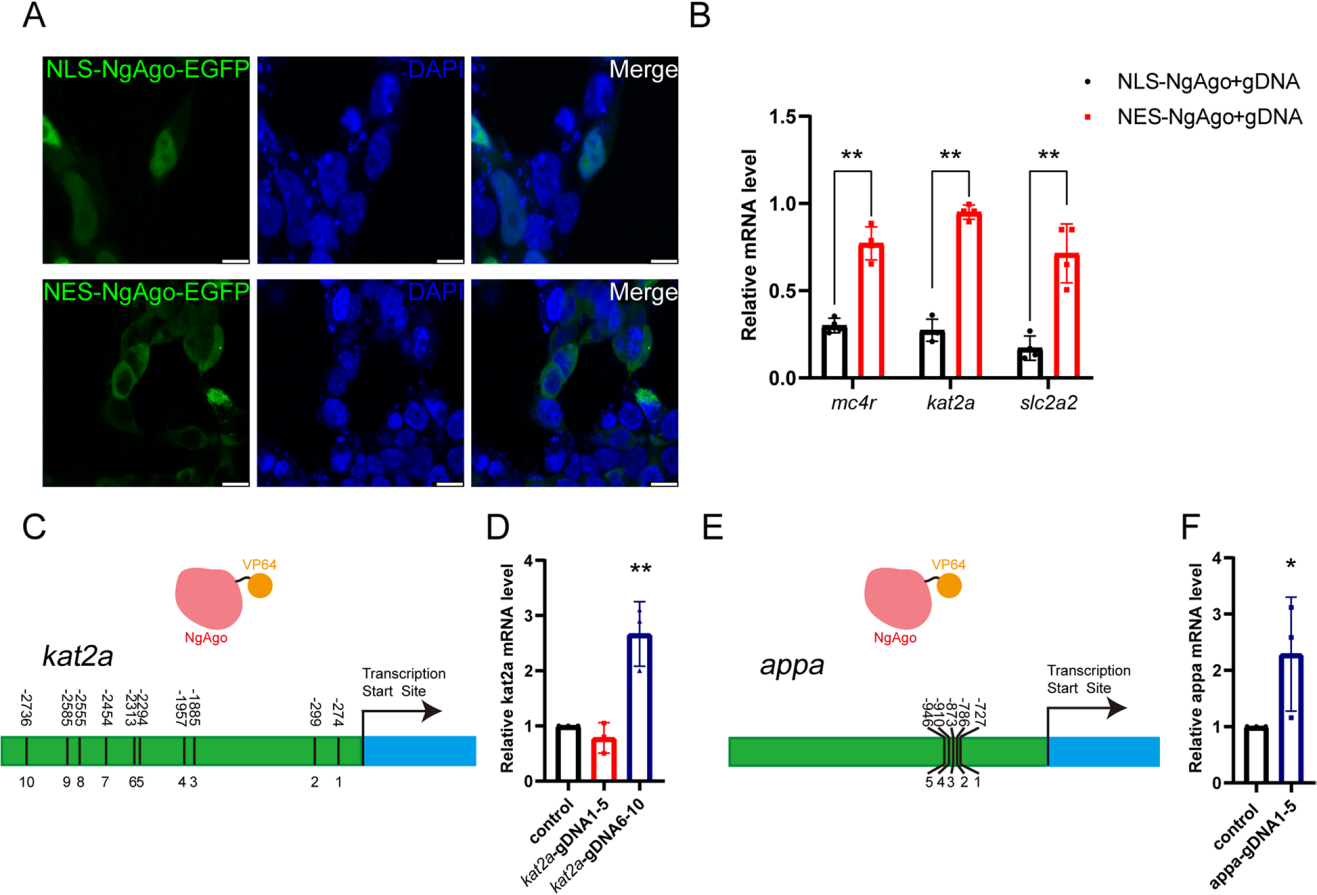
NgAgo targets genomic DNA in the nucleus. **A** Localization of NLS-NgAgo-EGFP and NES-NgAgo-EGFP in HEK293T cells. Scale bar = 10 μm. **B** Knockdown efficiency of NLS-NgAgo and NES-NgAgo on *mc4r*, *kat2a,* and *slc2a2*. Experiments were performed biologically four times. The bar displays mean ± se, and Student’s t test was used and the ** indicated the difference compared to control is statistically significant (*p* < 0.01). *n* = 4. **C**, **D** Upregulation of *kat2a* expression using NLS-NgAgo-VP64 guided by gDNAs. *n* = 3. **E**, **F** Upregulation of *appa* expression using NLS-NgAgo-VP64 guided by gDNAs. *n* = 4. The qRT‒PCR experiments were performed biologically three times. The bars display mean ± se, and Student’s t test was used and the ** and * indicated the difference compared to control is statistically significant (*p* < 0.01 and *p* < 0.05, respectively)

We’ve been using an NgAgo construct that contains nuclear localization signals (NLS) flanking NgAgo [12], and we then constructed nuclear export signal (NES)-NgAgo to see if our previous observations resulted from NgAgo functioning in the nuclei. Both NLS-NgAgo-EGFP and NES-NgAgo-EGFP were transfected into HEK293T cells and allowed to be expressed for 48 h before imaging. NLS-NgAgo-EGFP was mainly detected in the nuclei, and NES-NgAgo-EGFP was mainly in the cytoplasm (Fig. 4A). When coinjected with gDNAs, NES-NgAgo was not as efficient in downregulating the target genes as NLS-NgAgo (Fig. 4B, Additional File 13: Table S11). This is consistent with the possibility that NgAgo/gDNA targets genomic DNA rather than nucleic acid molecules in the cytosol. However, a readout responsive only to the binding of NgAgo to DNA is essential to prove this.

We thus constructed a plasmid to express an NgAgo-VP64 (four repeated VP16 fragments in tandem) fusion protein, which should activate gene expression if it sits on the promoter of a target gene. We coinjected NgAgo with five gDNAs for the promoter of *kat2a* (Fig. 4C, Additional File 14: Table S12). Interestingly, one set of gDNAs (gDNAs 6 to 10) was significantly more effective in upregulating target gene levels than the other (gDNAs 1 to 5) that was more proximal to the TSS (Fig. 4D, Additional File 15: Table S13). We inferred that gDNAs 1 to 5, or some of them, were too close to the transcription start site and hindered the binding of RNA polymerase to DNA and therefore failed to increase the expression level of *kat2a* mRNA. We designed five gDNAs for *appa* (Fig. 4E, Additional File 14: Table S12), which were targeted to ~ 800 bp upstream of the TSS, and they also effectively increased the mRNA level of *appa* (Fig. 4F, Additional File 16: Table S14).

Overall, the effectiveness of sense gDNAs in downregulating mRNA levels and NgAgo-VP64 in upregulating gene expression suggests that NgAgo/gDNA binds to genomic DNA, which means NgAgo-gDNA may be utilized to manipulate gene expression at the transcription level, while the effective downregulation of mRNA levels by gDNA targeting exon‒exon boundaries suggests binding of NgAgo/gDNA to mRNA.

## Discussion

Gene knockdown and gene knockout methods are utilized to manipulate gene expression for studies of gene function and clinical applications. The Argonaute-like protein NgAgo was originally believed to be a DNA endonuclease. When we tested it in a zebrafish model, we found that NgAgo could reduce RNA levels without introducing indel mutations in DNA. In this study, we used zebrafish embryos as a model to test NgAgo on numerous genes and confirmed that NgAgo can be used on various genes to downregulate gene expression and observed some phenotypes consistent with previous reports.

To further improve NgAgo-mediated knockdown, we analyzed some influential factors. Our data supported the idea that the knockdown efficiency of NgAgo is related to the GC content of the gDNA and the relative position of the target site. When analyzing individual genes, when the GC content of gDNA was greater than 50%, we could expect a higher chance of successfully downregulating the expression of the target gene. This was possibly due to the longer binding of the NgAgo/gDNA complex to its target caused by stronger hydrogen bonds. Regarding the position of the gDNA target site, we found that gDNA targets close to the transcription start site are associated with a stronger effect of downregulation, while on gDNA targets at the tail of genes, it was highly unlikely to obtain effective downregulation. These observations are similar to what was reported for CRISPRi, which works most effectively when targeted proximal to the TSS [21].

In addition, when transfected into cells, NgAgo-EGFP amounts in the nuclei of cells varied dramatically after transfection. Similarly, microinjected NgAgo mRNA and gDNA are unevenly distributed in the cells of an embryo, and the developing zebrafish embryos might compensate for a minority of cells being eliminated or compromised when an essential gene is targeted. Making NgAgo/gDNA more uniformly available to all cells should increase its efficiency.

In this study, we studied some factors (including GC ratio, target position, 5’ phosphorylation, and targeted strand) of gDNA affecting gene downregulation. We noticed that NgAgo/gDNA downregulates gene expression when targeted to the transcribed region, and gDNAs targeting the 5’ part and the 3’ part can both be effective, which is different from CRISPRi, which is functional when targeted to a locus very proximal to the TSS. Based on the evidence of NgAgo-VP64 with gDNAs targeting promoters upregulating the target genes, we suggest that the NgAgo/gDNA-based downregulation can be explained by the working model of NgAgo-gDNA binding to the target genomic DNA loci and blocking the extension of RNA polymerase. Thus, we conclude that NgAgo-VP64/gDNA shares a working model with CRISPRa, and NgAgo/gDNA primarily works similarly to CRISPRi [22].

## Conclusions

**Fig. 5:**
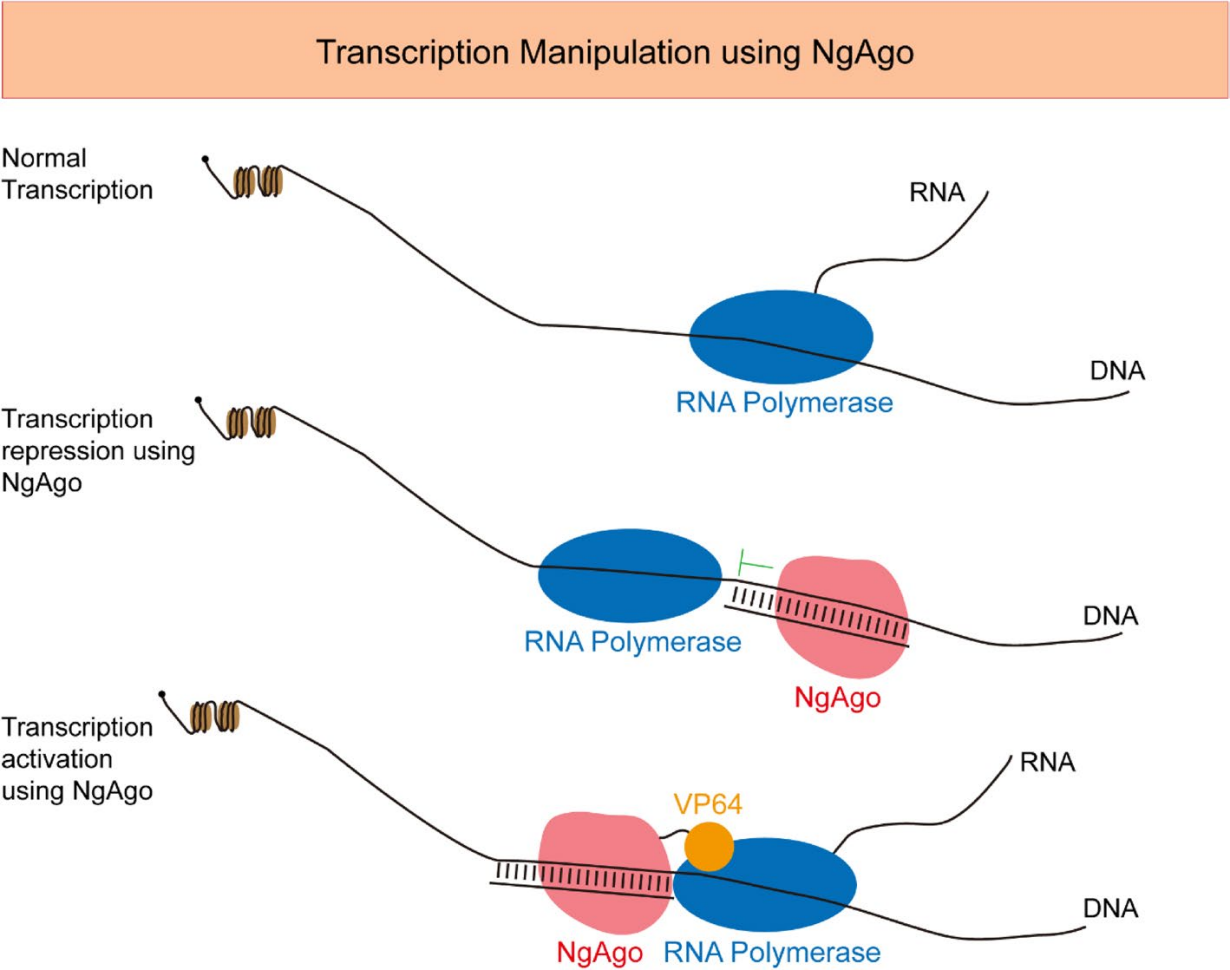
Working models of NgAgo-gDNA repression of transcription and NgAgo-VP64 activation of transcription. Upon NgAgo-gDNA binding to a gene, RNA polymerase progression is blocked, reducing the transcript level of the targeted gene. Recruitment of RNA polymerase by four tandem VP16s (VP64) fused to NgAgo can be used to activate target gene expression

Overall, this study demonstrates that NgAgo can be used for the manipulation of gene expression and provides insights into factors influencing the efficiency of such applications (Fig. 5). The efficiency remains to be improved before it can be widely used for downregulating genes in zebrafish, in which most genes are haplo-sufficient. Further studies on the potential DNA-nicking activity of NgAgo in eukaryotic cells and any possible modifications to boost its transcriptional activation/repression efficacy are required in the future.

## Methods

### Zebrafish husbandry

Zebrafish (Tübingen strain) were provided by the Zebrafish Facility at Nantong University under standard laboratory conditions of 14 h light/10 h darkness at 28.5 °C. Embryos were collected and kept in E3 buffer containing 0.17 mM KCl, 5.03 mM NaCl, 0.33 mM CaCl_2_·2H_2_O, and 0.33 mM MgSO_4_·7H_2_O in H_2_O. All the studies reported here were submitted to the Ethics Committee on Animal Experimentation of Nantong University, and all procedures were approved by the Animal Care and Use Committee of Nantong University.

### Bioinformatics

The genomic and mRNA sequences in Supp. Table 2 were obtained from GenBank. Sequences were analyzed in Vector NTI (Thermo Fisher). gDNAs were manually designed.

### gDNA preparation and synthesis

gDNAs were synthesized as DNA oligos by Genscript (Nanjing, China).

### Morpholino oligonucleotides and microinjection

The MOs were synthesized by Gene Tools. MO antisense oligomers were prepared at a stock concentration of 1 mM according to the manufacturer’s protocol. The sequence of zebrafish *pax6a* splicing MO in this study was 5’-acggagcacaggtattctcctcacc-3’, *fignl2* splicing MO was 5’-tcagaaatgtagcacttactatagg-3’, and the standard control MO was 5’-cctcttacctcagttacaatttata-3’. NgAgo mRNA was generated using the mMessage mMachine SP6 Kit (Ambion) with the pCS2-NLS-NgAgo plasmid template [12]. Each embryo received approximately 1 nL of the mixed solution of NgAgo mRNA and gDNA using borosilicate glass capillaries with an IM-400 Electric Microinjector (Narishige, Japan) at a dose of 0.34 ng/embryo of NgAgo mRNA and 0.1 pmol/embryo of gDNA. The injected embryos were grown and harvested at 12 hpf or 24 hpf for RNA isolation.

### RNA extraction, reverse transcription, and qRT‒PCR

In brief, zebrafish embryos were homogenized in a 1 mL TransZol Up Plus RNA Kit (TransGen Biotech, China), and RNA isolation was performed according to the manufacturer’s instructions. One microgram of RNA was reverse-transcribed into cDNA using a HiScript III 1st Strand cDNA Synthesis Kit (+ gDNA wiper) (Vazyme, China). Synthesized cDNA was stored at -20 °C. qRT-PCRs were run on an ABI Step One instrument in a total volume of 20 μL using the primers listed in Supplementary Table 2, and the reference gene was *18S rRNA*. The data shown are from at least three batches of injected zebrafish embryos, each containing 20 to 30 embryos, and the qRT*‒*PCRs were run in three technical replicates. Primers are listed in Additional File 17: Table S15.

### Imaging

Embryos were placed in 0.16 mg/ml tricaine and imaged with an Olympus SZX16 stereo microscope.

### Statistics

Data are expressed as the mean ± se (standard error of the mean). All experiments in this study were repeated biologically three times or more. Data were analyzed in GraphPad Prism 8 (http://www.graphpad.com/scientific-software/prism/). All primers were designed using Primer Premier 5. Statistical analysis was performed using one‐way analysis of variance (ANOVA) for comparisons among genotype groups followed by Student's *t* test for comparisons between two groups. A *p* < 0.05 is considered statistically significant.

## Supplementary Information

**Additional file 1: Table S1.** Sequences and knockdown efficiency of gDNAs used in Figure S1.

**Additional file 2: Figure S1.** 5’ phosphorylation does not affect NgAgo/gDNA effectiveness, while target position and GC ratio contribute to the efficiency of gene knockdown.

**Additional file 3: Table S2.** Survival after injection of NgAgo mRNA and gDNA, associated to Fig. 1A.

**Additional file 4: Table S3.** Relative mRNA levels of genes targeted by NgAgo-gDNA in Fig. S1A.

**Additional file 5: Table S4.** Relative expression level of kat2a slc2a2 pre-mRNA targeted by NgAgo-gDNA, associated to Fig. 2B.

**Additional file 6: Table S5.** Fig. 2B kat2a slc2a2 mRNA relative expression level. Relative expression level of kat2a and slc2a2 mRNA targeted by NgAgo-gDNA, associated to Fig. 2B.

**Additional file 7: Table S6.** Relative mRNA level of kat2a targeted with NgAgo-gDNA of various GC ratio, associated to Fig. 2C.

**Additional file 8: Table S7.** Relative mRNA level of slc2a2 targeted with NgAgo-gDNA of various GC ratio, associated to Fig. 2D.

**Additional file 9: Figure S2.** Full gel image of Fig. 3B.

**Additional file 10: Table S8.** Fig. 3E fignl2. Relative fignl2 mRNA level after injection of NgAgo-gDNA.

**Additional file 11: Table S9.** Sequences of gDNAs targeting exon-exon boundaries or introns in mRNA, associated with Fig. 3FG.

**Additional file 12: Table S10.** Relative mRNA levels of genes after injection of NgAgo-gDNAs targeting exon-exon boundaries or introns in mRNA, associated with Fig. 3FG.

**Additional file 13: Table S11.** Fig. 4B NLS NES qPCR. Relative mRNA level of target genes targeted by NLS-NgAgo+gDNA or NES-NgAgo+gDNA.

**Additional file 14: Table S12.** Sequences of gDNAs targeting promoters of kat2a and appa.

**Additional file 15: Table S13.** Relative expression level of kat2a mRNA after injection of NgAgo-VP64+gDNAs, associated with Fig. 4D.

**Additional file 16: Table S14.** Relative expression level of appa mRNA after injection of NgAgo-VP64+gDNAs, associated with Fig. 4F.

**Additional file 17: Table S15.** qRT-PCR primers used in this study.

## Data Availability

All data generated or analyzed during this study are included in this published article and its supplementary information files (Additional Files 1, 2, 3, 4, 5, 6, 7, 8, 9, 10, 11, 12, 13, 14, 15, 16 and 17).

## Abbreviations

NgAgo: *Natronobacterium gregoryi* Argonaute
gDNA: Guide DNA
CRISPR/Cas9: Clustered regularly interspaced short palindromic repeats/CRISPR-associated protein 9
CRISPRi: CRISPR interference
EGFP: Enhanced green fluorescent protein
MO: Morpholino oligonucleotides
RT-qPCR: Reverse transcription-quantitative Polymerase Chain Reaction
PfAgo: *Pyrococcus furiosus* Argonaute
TtAgo: *Thermus thermophilus* Argonaute
TSS: Transcription start site
NLS: Nuclear localization signal
NES: Nuclear export signal
