# Supplementary figures and images for: Efficient manipulation of gene expression using Natronobacterium gregoryi Argonaute in zebrafish
Source: BMC Biol. 2023 Apr 24;21:95. doi: 10.1186/s12915-023-01599-x (PMC10127001; doi:10.1186/s12915-023-01599-x)

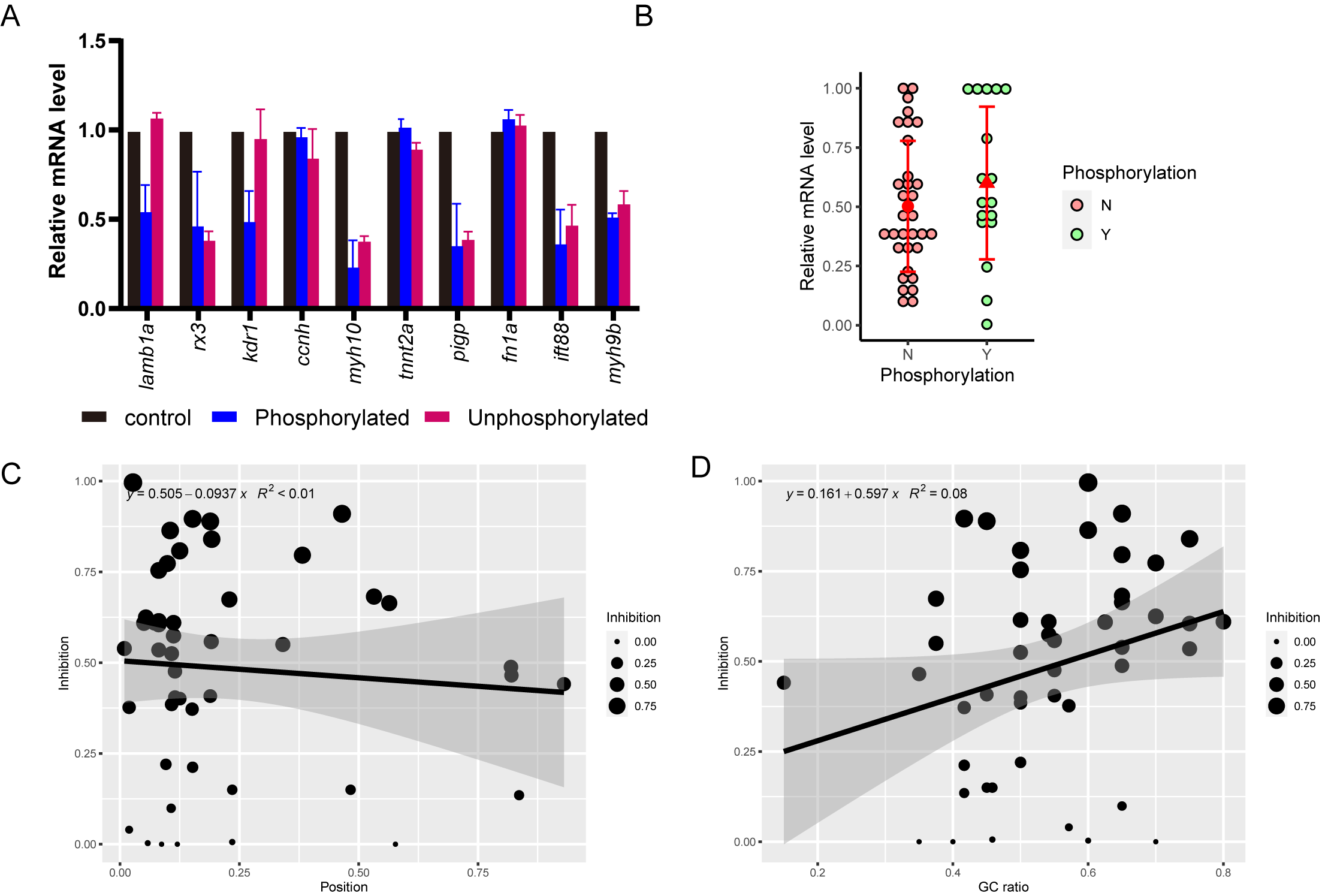

Supplement: Supplementary file 2 — Additional file 2: Figure S1. 5’ phosphorylation does not affect NgAgo/gDNA effectiveness, while target position and GC ratio contribute to the efficiency of gene knockdown. [file 12915_2023_1599_MOESM2_ESM.tif]

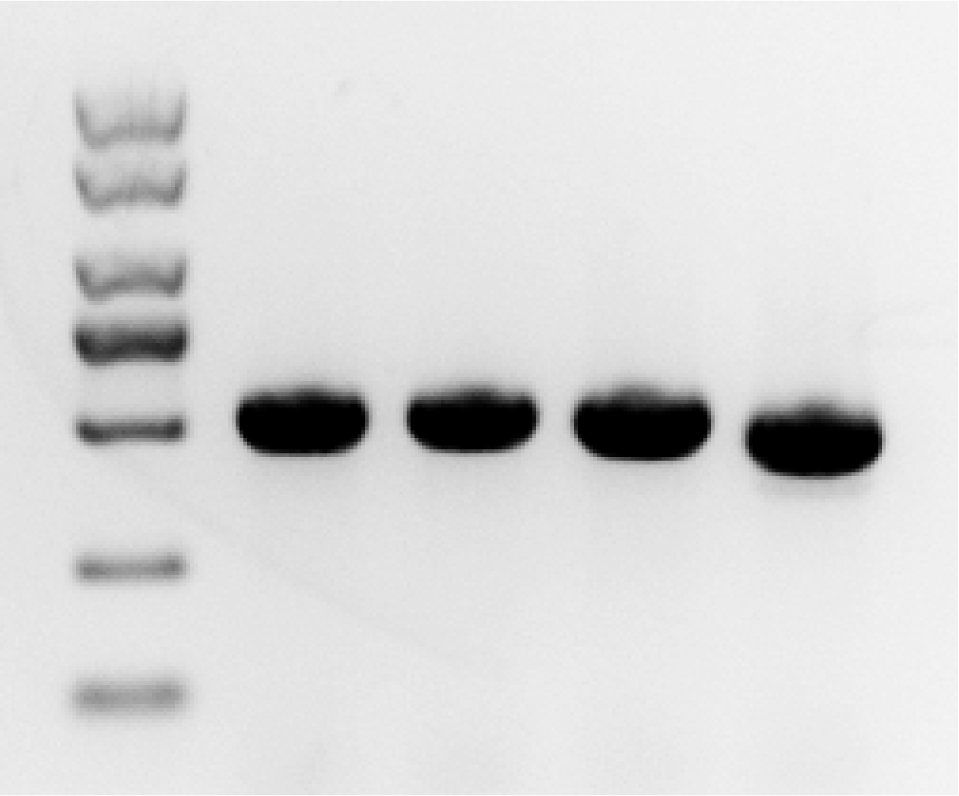

Supplement: Supplementary file 9 — Additional file 9: Figure S2. Full gel image of Fig. 3B. [file 12915_2023_1599_MOESM9_ESM.tif]
